# Understanding the Cap Structure in K2P Channels

**DOI:** 10.3389/fphys.2016.00228

**Published:** 2016-06-14

**Authors:** Leandro Zúñiga, Rafael Zúñiga

**Affiliations:** Escuela de Medicina, Centro de Investigaciones Médicas, Universidad de TalcaTalca, Chile

**Keywords:** K2P channels, two-pore domain channel, cap structure, K2P potassium channels structure, potassium channels

The two-pore domain potassium (K2P) channel family is composed by 15 members, identified in the human genome, and also K2P channels have been identified in yeast, plants, zebrafish, nematode and fruitfly (Goldstein et al., 2001). Based on their primary structure and functional properties, K2P channels are grouped into six distinct subfamilies denoted as TREK, TALK, TASK, TWIK, THIK, and TRESK (Goldstein et al., 2001, 2005; Figure 1A).

**Figure 1:**
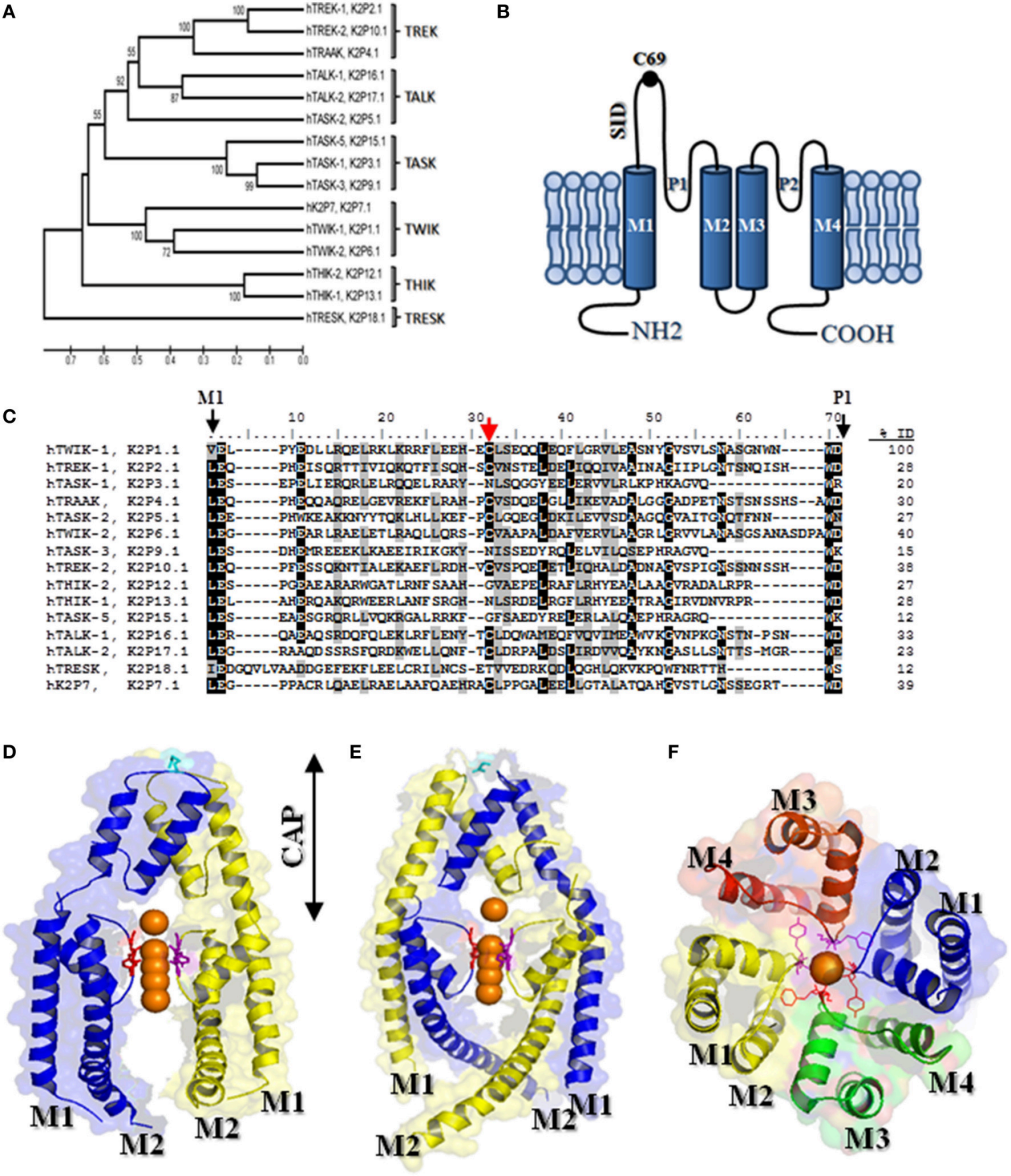
**K2P channels: (A)** Phylogenetic tree for K2P family (the phylogenic analysis was done with MEGA software version 5). The length of lines indicates the relative distances between nodes and it is scaled below the tree. Numbers on branches indicate bootstrap values (as a percentage). **(B)** Topological model proposed for K2P channels, each subunit has two pore forming domain (P loops) and four transmembrane domains (denoted M1-M4). **(C)** Amino acid sequence alignment of K2P channel M1-P1 domain. Gaps are indicated by dashes, letters with black background are identical amino acids, and letters with gray background are similar amino acids. Red arrow indicates the cysteine residues involved in the formation of covalent bridge between two subunits. Amino acid identity (ID) among the M1-P1 domains of hTWIK-1 and the others K2P subunits was calculated. **(D)** View of the TRAAK structure (PDB code 3UM7) in ribbon representation, showing a non–swapped configuration (conventional configuration). **(E)** View of the TRAAK structure (PDB code 4I9W) in a domain swapped configuration. The potassium ions are colored orange and the chains are in blue and yellow. The disulfide bridge at the top of the cap is shown in stick representation colored cyan. **(F)** View from the extracellular side showing the TRAAK pore (PDB code 3UM7). The GYG sequence of each P domain is highlighted in stick representation and the helical cap was removed. For clarity in **(D,E)**, the second pore domain was removed from each TRAAK subunits.

K2P channels play several key roles in excitable cells. For instance, K2P channels generate the called leak or background currents that are responsible for the maintenance of the resting membrane potential close to the equilibrium potential of K^+^ (E_K_) which sets the resting membrane potential below the firing threshold (Lesage and Lazdunski, 2000; Goldstein et al., 2001; Lotshaw, 2007; Enyedi and Czirjak, 2010).

K2P channels have also been linked to several pathologies. For example, TASK-1 malfunction as consequence of missense mutations or its pharmacological inhibition has been associated to pulmonary hypertension (Ma et al., 2013) and cardiac arrhythmias (atrial fibrillation) (Schmidt et al., 2015), respectively. Additionally, mutations of TASK-3 (G236R) and TRESK are linked with the Birk Barel syndrome (Barel et al., 2008) and familial migraine (Lafreniere et al., 2010). On the other hand, an overexpression of TASK-3 has been found in some human breast cancer tumors where it has been proposed acts as a proto-oncogene (Mu et al., 2003). For comprehensive reviews see Enyedi and Czirjak (2010) and Feliciangeli et al. (2015). Taken together, K2P channels could be potential pharmacological targets for restoring the function of dysfunctional excitable tissues as well as in cancer treatment.

The open probability of K2P channels is highly regulated by a wide variety of stimuli such as kinases, phosphatases, lipids, G proteins, internal and external pH, mechanical force, protein-protein interactions and volatile anesthetics (Lesage and Lazdunski, 2000; Goldstein et al., 2001; Lotshaw, 2007; Enyedi and Czirjak, 2010).

K2P channels display a structure that markedly differs to that described for Kv and Kir K^+^ channel families. Each K2P subunit consists of four transmembrane domains (M1-M4 domains), two pore forming domains (P domains) with both N- and C- termini facing the cytosolic side (Figure 1B). Given that 4 P domains are required to form a K^+^ selectivity filter, it was assumed that the K2P channels must function as dimers. Strong evidence to support this architecture was initially reported (Lesage et al., 1996; Lopes et al., 2001) and then it was corroborated by crystallographic structures obtained from K2P members (Brohawn et al., 2012; Miller and Long, 2012) (see Figure 1F).

Another particular feature of K2P channels is the so-called cap structure that is formed by the extracellular M1-P1 linkers of each K2P monomer (Brohawn et al., 2012; Miller and Long, 2012). The elucidation of the crystal structure of TWIK-1 and TRAAK channels showed that the cap forms two tunnel-like side portals which serve as extracellular ion pathway (Brohawn et al., 2012; Miller and Long, 2012; Figure 1D).

Two structures recently solved at very high resolution confirmed the presence of the cap structure in TRAAK and TREK-2 channels (Brohawn et al., 2013; Dong et al., 2015). Nevertheless, a closer look at the cap structure revealed that the disposition of the helices (two per monomer, denoted as inner and outer helices, respectively) forming the cap structure in TRAAK and TREK-2 differs from that originally reported for TWIK-1 and TRAAK channels (Brohawn et al., 2013; Dong et al., 2015). The structural difference is based on whether the outer helix from each M1-P1 monomer interacts with the inner helix from the same (conventional configuration, Figure 1D) or the other monomer (swapped configuration, Figure 1E).

Little is known about the functional role and the molecular determinants responsible for the maintenance of the cap structure. The first study assessing in this matter by Lessage et al. showed that the M1-P1 linker form a particular coiled-coiled domain in TWIK-1 channels, where the two cysteine residues in position 69 from each monomer form an inter-chain disulfide bridge (Lesage et al., 1996). Moreover, the mutation of the cysteine residue in position 69 (C69) prevented the formation of covalent dimers and resulted in a loss of function in TWIK-1 channels, suggesting that the M1-P1 linker plays an essential role in the channel function (Lesage et al., 1996). This particular domain was called by Lessage and co-workers as SID for self-interacting domain (Figure 1B). The SID domain is conserved in all K2P channels (Figure 1C). The presence of covalent dimers was then corroborated in native tissue for TWIK-1, TREK-1 and TRAAK channels (Cluzeaud et al., 1998; Maingret et al., 2000; Reyes et al., 2000). The covalent dimerization has been also corroborated for recombinant TASK-2 and KCNK7 channels (Salinas et al., 1999; Lesage et al., 2001).

Further sequence analysis of K2P channels showed that the cysteine residue equivalent to the C69 is not conserved in other members of the family of K2P channels (subfamilies TASK and THIK) (see Figure 1C). However, a recent work by Goldstein et al. provides evidence for the presence of a cap structure in K2P channels that do not have a cysteine residue in its M1-P1 linker (Goldstein et al., 2016). Additionally, the mutation of the homologous cysteine to serine in the M1-P1 linker neither affected TASK-2 function (Niemeyer et al., 2003) nor the dimer formation of TWIK-2 and TREK-1 channels (Patel et al., 2000; Hwang et al., 2014). Together, the evidence exposed above suggest that the cysteine residue located in M1-P1 linker is not relevant for the formation and/or maintenance of the cap structure or the cap structure does not play a critical role in the function of K2P channels. However, the presence of the C69 in the SID domain is necessary to the covalent binding of interacting K2P subunits and potentially increases the stability of the dimers. More structural data from the mutants described above would provide clues to the understanding of the importance of the cap structure to the channel function. The article published by Goldstein et al. studied by alanine-scanning mutagenesis the effect of mutations in the M1-P1 linker on TASK-1 activity (Goldstein et al., 2016). The data obtained in this screening was then used to build a model of the TASK-1 cap that showed a strong structural similarity to that reported by x-crystallography for channels displaying a cysteine residue in the M1-P1 loop (Goldstein et al., 2016). Interestingly, the model showed that the M1-P1 linkers from each monomer form a coiled coil domain. Coiled-coils consist of two to five amphipathic α-helices that twist around one another to form a supercoil (Burkhard et al., 2001). In the case of K2P channels, two α-helices form a parallel left-handed coiled coil that is characterized by a seven-residue periodicity (heptad repeat), with the occurrence of apolar residues preferentially in the first (a) and fourth (d) positions of the “heptad” (Burkhard et al., 2001). Consistent with such a particular domain in TASK-1, a severe loss of function was observed in TASK-1 upon mutation of residues L48, Y52, L54, S55, Y59, and L62 which are from “a” and “d” sites that are critical for the coiled-coil formation (Goldstein et al., 2016).

Little is known about the role of the cap structure in K2P channels but it has been shown in many coiled coil-displaying proteins that this domain plays an essential role in oligomerization (Burkhard et al., 2001). Hence, it is plausible that the cap structure may play a critical role in K2P dimerization. On the other hand, there is also evidence for the importance of the cap structure in K2P channel activity (Morton et al., 2005; Gonzalez et al., 2013) and the insensitivity of K2P channels to classical K^+^ blockers (e.g., TEA, 4AP or toxin), precluding the blockers access to the pore (Brohawn et al., 2012; Miller and Long, 2012).

Another equally interesting feature is that the K2P channels are able to assemble heterodimers in different tissues, where they account for a significant part of the native leak current. For example, TASK-1/TASK-3 heterodimers play an important role in chemoreception, acting as primary sensors of hypoxia and metabolic acidosis (Buckler et al., 2000; Czirjak and Enyedi, 2002; Berg et al., 2004). TWIK-1 may also interact with TASK-1 and TASK-3 subunits in the cerebellum (Plant et al., 2012). TWIK-1/TREK-1 heterodimers generate passive conductance and cannabinoid-induced glutamate release in astrocytes (Hwang et al., 2014). The THIK-2/THIK-1 heterodimers contribute to cell excitability, rescuing the silent THIK-2 subunit (Blin et al., 2014). And recent studies have shown the heterodimeric assembly of members from the TREK subfamily (Blin et al., 2016; Levitz et al., 2016).

These heterodimers configurations present properties different from those observed in their corresponding homodimers, increasing the K^+^ channel diversity. Thus, the K2P subunits of the same or distant subfamilies that show an overlapping tissue distribution might form heterodimers and produces active channels. Perhaps the new cap structures present in these heterodimeric configurations could affect the gating mechanism and, at least in part, explain the new properties.

In summary, the cap structure in K2P channels seem to be conserved but the function of the cap structure and the mechanism involved in its formation and maintenance might differ among the K2P members. The elucidation of other cap structures of other K2P channels might get clues into the functional implications of conventional vs. swapped cap structures. It is tempting to speculate that the configuration of the cap structure might confer unique features in K2P channels that have the ability to form functional heterodimeric complexes such as those formed by TWIK1/TASK-1/TASK-3 (Buckler et al., 2000; Czirjak and Enyedi, 2002; Berg et al., 2004; Plant et al., 2012), TWIK-1/TREK-1 (Hwang et al., 2014), THIK-2/THIK-1 (Blin et al., 2014) or TRAAK/TREK1/TREK2 (Blin et al., 2016; Levitz et al., 2016).

## Author contributions

All authors listed, have made substantial, direct and intellectual contribution to the work, and approved it for publication.

### Conflict of interest statement

The authors declare that the research was conducted in the absence of any commercial or financial relationships that could be construed as a potential conflict of interest.
